# Intramedullary Osteosclerosis of the Tibia: A Rare Cause of Osteosclerosis to Keep an Eye On

**DOI:** 10.7759/cureus.36873

**Published:** 2023-03-29

**Authors:** Alaa Al-Taie, Aisha Al-Boinin, Ahmed Elmustafa Musa

**Affiliations:** 1 Radiology, Hamad General Hospital, Doha, QAT

**Keywords:** imaging, musculoskeletal radiology, tibia sclerosis, orthopedics, osteosclerosis

## Abstract

Intramedullary osteosclerosis (IMOS) is a rare process of intramedullary bone formation in one or more bones in the lower limb. It arises in adulthood, its etiology is unknown, and it is diagnosed by exclusion. We present a case of a 26-year-old female who presented with three-week history of right tibial pain provoked by prolonged standing. Imaging revealed sclerotic cortical thickening, in addition to sclerosis at the mid-tibial segment. Intramedullary osteosclerosis is an uncommon cause of leg pain. The literature revealed that non-steroidal anti-inflammatory drugs (NSAIDs) might be of use to help ease the pain.

## Introduction

Intramedullary osteosclerosis (IMOS) was first described in 1988 by Abdul-Karim et al. [1]. It is also known as monomelic medullary osteosclerosis [2], a term that describes an uncommon process of intramedullary bone formation in one or more bones in the lower limb, most commonly in the mid-tibia [3], followed by the femur [4].

Its cause is unknown; there is no association with family history, trauma, or organic pathological causes [5]. It has no particular etiology and presents as chronic severe pain, with partial/complete relief on non-steroidal anti-inflammatory drugs (NSAIDs) and difficulty ambulating in some cases [4]. Findings on plain radiography and computed tomography (CT) scans are of a massive diaphyseal osteosclerotic lesion. In addition to that, laboratory investigations are typically unremarkable [3,6]. Furthermore, it has a benign radiographic appearance; it appears without extensive new bone formation and soft tissue abnormality, unlike malignancies, for example, osteosarcoma and lymphoma.

It is an adult-onset condition, positively related to being a female, and not related to personal or family history, and it has an asymmetric skeletal distribution [3,7]. No diagnostic criteria or treatment is currently established; therefore, a delay in diagnosis is not uncommon [7].

Intramedullary osteosclerosis is a rare condition and diagnosed by exclusion; it is diagnosed after ruling out other possible conditions [1,3]. Differential diagnoses include hereditary multiple diaphyseal sclerosis, malignancy, osteomyelitis, melorheostosis, and stress fracture [1,8].

## Case presentation

Our patient is a 26-year-old female who presented with three weeks of pain in the right tibia after prolonged periods of standing. She first experienced the pain while walking but then reported that extended standing sessions also elicited the pain. However, she did not feel pain when resting. She also did not recall any trauma, insect bites, rashes, or systemic symptoms. Her past medical history was clear, and she generally led a normal life. History was otherwise unremarkable. On examination, there was local tenderness to palpation of the anterior and medial aspect of the mid-right leg without erythema or swelling. She could bear weight normally, and her neurovascular status was intact. Pain is particularly located at the anterior aspect of the mid-tibia. Physical examination showed mild swelling. The patient was discharged on analgesia (non-steroidal anti-inflammatory medications) and instructed to adjust her physical activity according to her pain tolerance. She was also booked for an X-ray.

**Figure 1:**
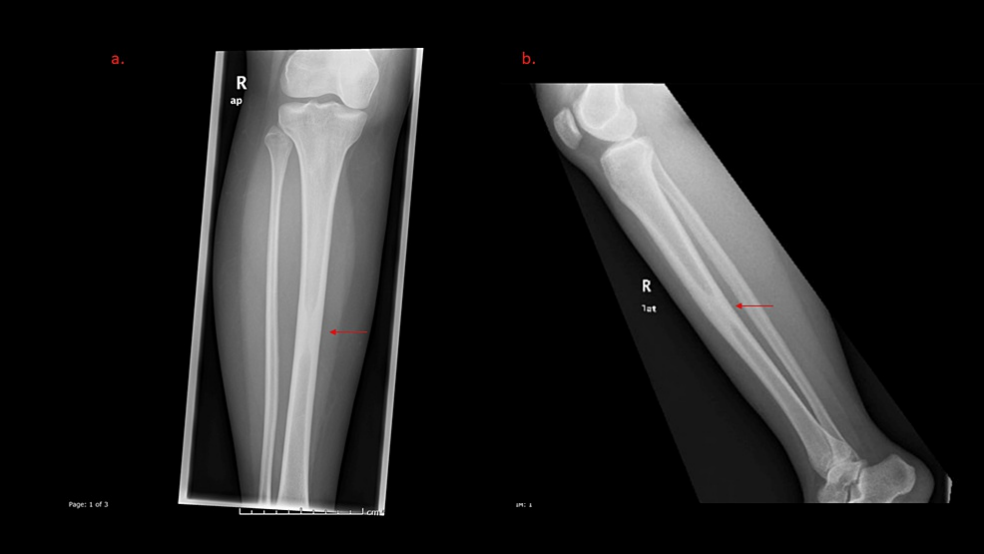
Frontal (a) and lateral (b) radiographs of the proximal tibia and fibula. Focal circumferential endosteal/intramedullary thickening and sclerosis involving the middle shaft of the tibia (arrows). It shows no destructive pattern, periosteal reaction, or soft tissue component.

X-ray showed a well-defined intramedullary sclerotic lesion of the mid-tibial shaft with a narrow zone of transition, and no fracture line was identified (Figure 1).

**Figure 2:**
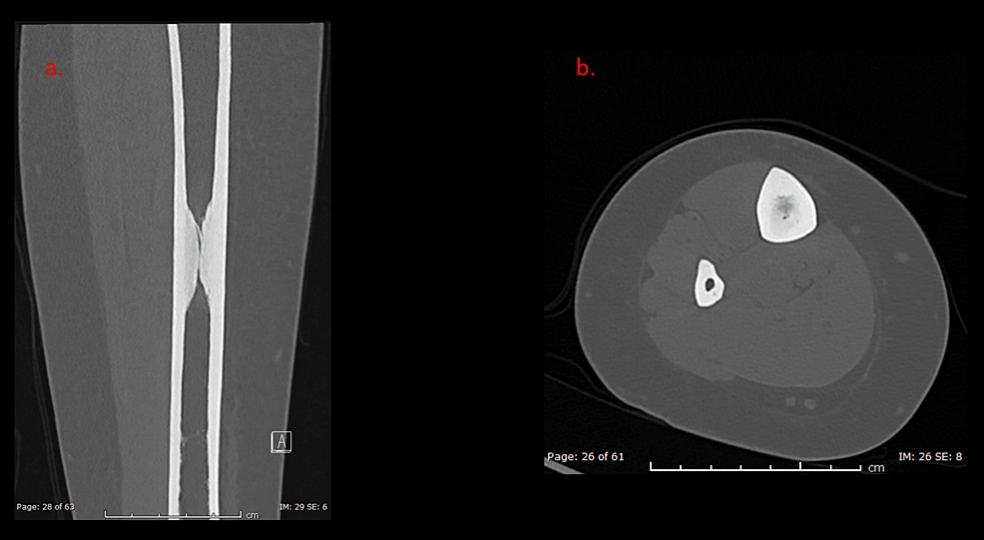
Coronal (a) and axial (b) computed tomography examination of the right leg in bone window. Focal circumferential endosteal/medullary solid cortical thickening of the mid-tibial shaft. No evidence of an old fracture line, cortical nidus or sequestrum, periosteal reaction, or soft tissue component.

Following the radiograph, the patient underwent a CT scan (Figure 2), which better illustrated the sclerotic lesion with medullary canal obliteration. There was no sinus, sequestrum, periosteal reaction, or soft tissue component. The patient did not recall any similar history in her family, and a skeletal survey showed no similar lesion elsewhere in her body.

## Discussion

Intramedullary osteosclerosis (IMOS) is a pathological condition with an etiology that is still unknown [5]. Clinically, the presence of pain without systemic symptoms favors this entity. However, this by itself is non-yielding.

In our case, the pain responded to non-steroidal anti-inflammatory drugs (NSAIDs), but the literature contains instances where NSAIDs were not helpful for the patient [4,7]. Pathologically, the condition demonstrates marrow replacement by variable degrees of increased trabeculation, sclerosis, and increased mineralization [3].

The role of the radiologist largely is excluding other differential diagnoses using a multi-modality approach if possible. These differential diagnoses include conditions where osteosclerosis is a feature, which includes sclerosing bone dysplasia, metabolic disorders, stress fractures, chronic osteomyelitis, and neoplastic process (osteoid osteoma, osteosarcoma, lymphoma, and osteoblastic metastases) [3-8]. Sclerosing bone dysplasia conditions have distinctive appearances in most of their types, the description of which is beyond the scope of this case report. Two conditions worth mentioning are Camurati-Engelmann and Ribbing diseases. Both are radiologically identical, but the former has an autosomal dominant inheritance and affects boys under 10 years who develop bone and muscle aches, abnormal gait, anemia, and symmetrical bilateral involvement of the tubular bones [9]. The latter, which is the closest in appearance to IMOS, can only be distinguished from it by its autosomal recessive inheritance and the absence of propensity to particular sex [10].

Metabolic disorders (renal osteodystrophy, pseudohypoparathyroidism, and pseudopseudohypoparathyroidism) are easily evaluated by their polyostotic involvement and altered blood chemistry. Additionally, diaphyseal periosteal new bone formation is noted in hypervitaminosis A [11]. The absence of characteristic symptomatology and a radiolucent nidus in computed tomography and radiographs effectively rule out osteoid osteoma [3-8]. Chronic osteomyelitis is corroborated by clinical history, examination, and laboratory findings of underlying infection [12]. It is unlikely to confer a malignant diagnosis from a purely medullary lesion without new bone formation and/or soft tissue component [4-8].

Abe et al. [7] and Chanchairujira et al. [3] described the utility of bone scans as a part of the diagnostic workup. They reported significant fusiform tracer uptake at the site of IMOS in the delayed phase. Abe et al. furthermore reported slightly increased uptake in the blood pool phase in one (out of three) of their cases. This helps differentiate IMOS from other entities such as stress fracture, osteomyelitis, and malignancy, where uptake in the vascular and blood pool phase increases.

No standardized management is currently being followed for IMOS. All reviewed literature utilized painkillers (NSAIDs) to alleviate the most common presenting symptom, including our patient, who got her symptoms controlled this way. Others [7,13-18] believed that intramedullary sclerosis results in increased intramedullary pressure, and by creating a hole or reaming of the lesion, the pressure will subside and improve symptoms. They reported alleviation of the symptoms between complete and partial (using less analgesia). However, the recurrence of similar or milder symptoms was associated with the healing of the medullary hole or reaming [7]. It is important to note that the reviewed literature for the aforementioned surgical management of this entity largely stems from scientific inquiry into closely similar conditions, namely, Ribbing disease [13-17] and Camurati-Engelmann disease [18]. According to Gaeta et al. [16] and Ziran et al. [17], additional medical management by bisphosphonates is unsuccessful.

## Conclusions

Intramedullary osteosclerosis is a rare entity of unknown etiology, with pain as the most common presenting symptom. The nonspecific appearance of this pathology renders our role as radiologists to exclude other causes of bone sclerosis before making the diagnosis. Non-steroidal anti-inflammatory medications are noted to improve symptoms in our patient, and then, follow-up can be arranged as necessary. However, further research into the subject is needed to reach a standard plan of care.
